# A proposed evaluation of new approach methodologies for dermal toxicity assessment of botanicals

**DOI:** 10.1080/13880209.2026.2717803

**Published:** 2026-08-18

**Authors:** Julie Krzykwa, Allison Hilberer, Karin Ames, Cécile Bascoul, WanYun Cheng, Rachel Doerer, Stefan Gafner, Dori Germolec, Hilva Gjoni, Shomit Nazare, Argel Islas-Robles, Çiğdem Kahraman, Isabelle Lee, Hans Raabe, Phillip Wages, Donna Webster, Hong Xie, Constance A. Mitchell, Gertrude-Emilia Costin

**Affiliations:** ^a^ Health and Environmental Sciences Institute, Washington, DC, USA; ^b^ Institute for In Vitro Sciences, Inc, Gaithersburg, MD, USA; ^c^ Procter & Gamble, UK; ^d^ dōTERRA, Pleasant Grove, UT, USA; ^e^ The Estée Lauder Companies Inc, Melville, NY, USA; ^f^ Amway Corporation, Ada, MI, USA; ^g^ American Botanical Council, Austin, TX, USA; ^h^ Division of Translational Toxicology, National Institute of Environmental Health Sciences, Durham, NC, USA; ^i^ University of Parma, Parma, Italy; ^j^ Department of Pharmacognosy, Faculty of Pharmacy, Hacettepe University, Ankara, Türkiye; ^k^ Research Institute for Fragrance Materials, Inc, Woodcliff Lake, NJ, USA; ^l^ Herbalife International of America Inc, Torrance, CA, USA; ^m^ US Food and Drug Administration, College Park, MD, USA

**Keywords:** Dermal toxicity, complex mixtures, NAMs, botanicals, dermal phototoxicity

## Abstract

**Background:**

Botanicals are widely used in topical formulations for cosmetics, hair care, and personal care products, reflecting consumer interest in plant-derived materials. Their complex and variable composition, presents challenges for traditional toxicological approaches developed for single chemicals. Although many botanicals are well tolerated, reports of skin irritation, sensitization, and phototoxicity highlight the need for reliable approaches to identify dermal hazards and evaluate risk. Established in silico, in chemico, and *in vitro* test methods, often referred to as New Approach Methodologies (NAMs), may offer efficient and mechanistically informed tools.

**Objective:**

The Dermal Working Group within the Botanical Safety Consortium (BSC) developed a strategy for evaluating existing NAMs for assessing dermal toxicity endpoints in botanicals, using literature evidence and data-rich botanicals to inform assay selection and anticipated performance. This narrative review describes the literature-based considerations used to select candidate assays and characterized reference botanicals for subsequent experimental evaluation.

**Methods:**

This narrative review describes considerations in the selection and application of *in vitro* and *in chemico* assays addressing skin irritation (OECD TG 439), phototoxicity (OECD TG 432), and skin sensitization (OECD TG 442 C, 442D, and 442E), together with *in silico* approaches.

**Results & Discussion:**

A set of reference botanicals spanning a range of expected outcomes, from botanicals not expected to have effects to known irritants, sensitizers, and phototoxicants, was chosen to provide a foundation for evaluating assay performance and identifying technical considerations.

**Conclusion:**

This manuscript describes the botanicals and assays being evaluated as part of the BSC’s planned dermal toxicity work. The goal of this work is to investigate the utility of NAMs for dermal safety evaluations of botanicals.

## Introduction

Botanicals are ingredients derived from whole plants, including plant parts such as roots, leaves, or flowers, fungi, or algae. These ingredients have been used in cosmetics for thousands of years (Pisanti et al. 2023). Today, consumer interest in natural ingredients, particularly plant-based materials, continues to grow, driving the widespread use of botanicals in modern personal care and cosmetic products. Products containing botanicals span a broad range of everyday items. This includes soaps, deodorants, shampoos, hair care and skin care products, makeup, shave creams, and toothpaste (Antignac et al. 2011; Ferreira et al. 2021). Unlike single chemicals, botanicals have a complex and variable chemical composition. This is due to multiple factors, including growing conditions, cultivar, plant part, and processing (*e.g.,* extraction and purification steps) (Bruni and Sacchetti 2009; Mitchell et al. 2022). For personal care products, botanicals can be utilized in several ways, such as ground plant material, plant extracts, waxes, lipids, essential oils, and purified plant compounds, which can alter their composition and, consequently, their safety profile (Antignac et al. 2011; Moore et al. 2020). The variability in the composition of samples from the same botanical species creates challenges in identifying a single representative sample for *in vivo* hazard testing. Additionally, evaluating multiple samples can require substantial resources (Prinsloo et al. 2017; Ryan et al. 2019). These challenges complicate the evaluation of botanical safety by standard toxicology methods. In addition, the overall biological activity of a botanical may not be predictable from its individual constituents alone, as interactions among constituents, including additive, synergistic, or antagonistic effects, as well as the presence of unidentified compounds, may influence the toxicity profile of the complete extract. While better understanding and studying the contribution of the constituents and mixture effects may be informative, the focus of the work is on whole substances and to evaluate the suitability of the NAMs to predict skin toxicity in humans.

While most botanicals do not cause safety issues, some have been reported to cause dermal toxicity. Exposure to poison ivy (*Toxicodendron radicans* (L.) Kuntze, Anacardiaceae), poison oak (*Toxicodendron pubescens* Mill., Anacardiaceae), and poison sumac (*Toxicodendron vernix* (L.) Kuntze, Anacardiaceae), which contain urushiol, are well known to result in allergic contact dermatitis (accordingly, these plant species are not used in personal care products). Topical use of tea tree (*Melaleuca alternifolia* (Maiden & Betche) Cheel, Myrtaceae) essential oil is relatively safe with minor adverse event reports. However higher concentrations can cause irritation, and oxidation products can be the cause of allergic reactions (Hammer et al. 2006). Some botanicals, particularly those from certain members of the citrus family, contain photosensitizing compounds called furanocoumarins. These can cause photoirritation upon activation by ultraviolet (UV) light (Grosu Dumitrescu et al. 2024). As consumers increasingly favor products with botanicals, there is a need to ensure their safety.

In the United States, cosmetics are regulated by the U.S. Food and Drug Administration (FDA). The Modernization of Cosmetics Regulation Act (MoCRA) expanded the FDA’s regulatory authority over cosmetic products to better ensure consumer safety (US FDA 2022). MoCRA establishes that companies that manufacture or market cosmetics bear legal responsibility for ensuring the safety of their products. The Act does not provide guidance on which safety tests or frameworks to use, allowing safety approaches to reflect current scientific standards, provided manufacturers can document the safety of finished products and ingredients. In parallel, the FDA has expressed support for the principles of replacement, reduction, and refinement (3Rs) and the advancement of scientifically valid non-animal approaches for safety assessment. In Europe, cosmetics must undergo a pre-market safety assessment that does not involve animal testing (European Commission 2013). While this paper is not intended to be a regulatory review, given the increasing use of botanicals in cosmetics and their inherent compositional variability, these regulatory frameworks create a need for standardized, non-animal methods to evaluate botanical safety.

The Botanical Safety Consortium (BSC) is a cross-sector initiative to evaluate whether existing new approach methodologies (NAMs), typically developed for single chemicals, are suitable for assessing botanicals as complex mixtures (Mitchell et al. 2022; Mitchell et al. 2025). This group comprises experts from academia, government, research organizations, and industry across various disciplines, including toxicology, pharmacognosy, and NAMs. While the BSC has historically focused on the oral route of exposure for dietary supplements, the Dermal Working Group is primarily focused on personal care products and cosmetics, as well as the dermal route of exposure.

This narrative review describes considerations in selecting assays for evaluation of their suitability for use with botanical extracts. To evaluate these existing tools, a set of reference botanicals were selected as case studies. These botanicals have either been documented to produce dermal toxicity or to have well-documented dermal safety and tolerability.

This manuscript describes the importance of dermal toxicity testing with considerations and potential application of currently available NAMs. A proposed testing strategy is considered for selected dermal safety endpoints to understand utility of test method application in evaluation of reference botanicals selected by the Working Group.

Ultimately, the information described will support the future assessment of assay suitability and aid in the development of a dermal botanical safety toolkit to guide hazard identification, prioritize further testing, and strengthen confidence in the safe use of botanicals in cosmetic and personal care products.

### Dermal toxicity

The skin serves as the body’s first line of defense against chemical exposure, playing a crucial role in maintaining homeostasis through the regulation of thermal, electrolyte, hormonal, metabolic, and immune processes. While it acts as a protective barrier, chemicals can still be absorbed through the skin, leading to systemic exposure. Additionally, toxicity can occur locally at the site of contact. Dermal toxicity refers to a substance’s ability to cause either a localized reaction or systemic toxicity upon skin contact (Chandra et al. 2015). The assays evaluated in the current work focus on localized reactions.

Skin irritation is a reversible reaction that can result from exposure to various substances. Historically, the Draize test in rabbits was used to assess dermal irritation (Draize et al. 1944). However, *in vitro* assays are now more commonly employed (see Section 2 below) due to a desire to reduce animal testing and increase human relevance. Irritation typically occurs following acute exposure. Some chemicals can also cause skin corrosion, though this is uncommon for botanicals.

UV radiation can induce phototoxicity, or photoirritation, when combined with exposure to certain chemicals that absorb UV light and transition to a higher-energy state. These substances can then generate reactive oxygen species (such as singlet oxygen and free radicals) or directly transfer energy to cellular components, leading to oxidative damage of proteins, lipids, and DNA present in the skin. Naturally occurring compounds such as furocoumarins (found in some members of the citrus [Rutaceae] and carrot [Apiaceae] families) are well-known examples of phototoxic agents (Hinton and Goldminz 2020; Hilberer et al. 2025).

Repeated exposure to certain chemicals can lead to skin sensitization, a delayed type IV hypersensitivity reaction. Historically, guinea pigs were used to assess sensitization (Momma et al. 1998). The test involved repeated administration of the test substance over several days/weeks, followed by a challenge at a nonirritating concentration after a rest period, to determine whether a hypersensitivity response occurred. Additionally, mice have been used to assess if chemicals can induce skin sensitization *via* the local lymph node assay (OECD 2010).

Overall, there is a move to conduct dermal toxicity without using animals, to address concerns about animal welfare and limitations associated with human relevance and reproducibility of animal-based results (Costa Gagosian et al. 2025; Raabe et al. 2025). This has led to the development of NAMs that are more relevant to humans.

## Selected assays for dermal toxicity

**Figure 1.**
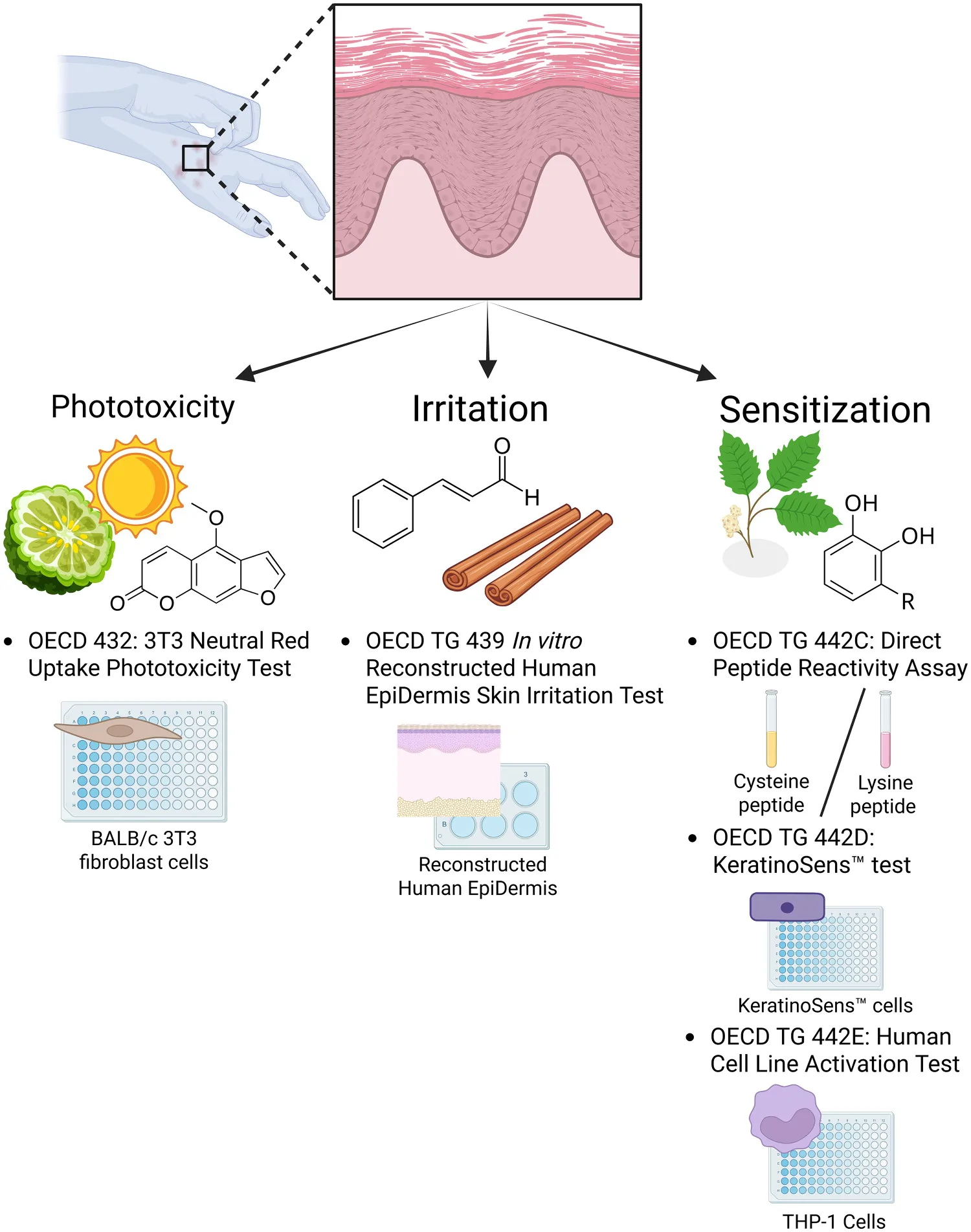
Overview of the selected NAMs used to evaluate selected dermal safety endpoints and their experimental set-up. Abbreviations: OECD, Organisation for Economic Co-operation and development; TG, test guideline; THP-1 cells, Tohoku Hospital Pediatrics-1 cells. Figure created in BioRender.

Given the mandate to move away from animal testing in some jurisdictions for cosmetics (European Commission 2009), dermal toxicity NAMs are more mature than other endpoints (*e.g.,* neurotoxicity), and several test methods have achieved regulatory acceptance as Organization Economic Co-operation and Development (OECD) Test Guidelines (TGs) or have been incorporated into the OECD Guidelines (GL; *e.g.,* the OECD 497 on defined approaches for skin sensitization (OECD 2025a)). As with other endpoints, a battery of assays is often needed to assess irritation, sensitization, and phototoxicity. The OECD TGs are regulatory-accepted, hazard-assessment tools for specific endpoints, and several of these TGs can be used for classification and labeling purposes (*e.g.,* the Globally Harmonized System [GHS]; United Nations 2023). Several test methods adopted as TGs were selected to evaluate if they can be suitable for hazard identification of botanicals (Figure 1), including skin irritation (OECD TG 439; OECD 2025a) phototoxicity potential (OECD TG 432; OECD 2019), and skin sensitization potential (OECD TG 442 C, OECD 442D, and OECD 442E, or more specifically the Direct Peptide Reactivity Assay (DPRA), KeratinoSens^™^ Assay, and human cell line activation test (h-CLAT), respectively; OECD 2024a; OECD 2024b; OECD 2025b). For skin irritation, the skin corrosion assay (OECD TG 431; OECD 2025d) is included in the Guidance Document on an Integrated Approach on Testing and Assessment (IATA) for Skin Corrosion and Irritation (OECD 2017); however, botanicals used in cosmetic applications are not generally expected to be skin corrosive because these ingredients have a long history of topical use and are not typically associated with the severe, irreversible tissue damage characteristic of corrosive substances. Instead, safety concerns for botanical ingredients more commonly relate to irritation, sensitization, phototoxicity, or systemic toxicity. Therefore, skin corrosion methods were not evaluated as part of the current Working Group efforts.

In the sections below, we briefly describe the currently selected tools, their overview and history, any experience reported with botanicals, and potential challenges and limitations. Our goal is to work with existing regulatory-based test methods and integrated testing approaches to determine assay performance, identify potential testing limitations, and inform future development of fit-for-purpose testing approaches. With movement toward the banning of animal testing in multiple jurisdictions, early evidence indicates some NAMs could be applicable to botanicals, though further evaluation for complex mixtures is needed (Puginier et al. 2022).

The assays selected for this work have undergone extensive international validation and regulatory review prior to adoption as OECD Test Guidelines. Reported performance characteristics, including sensitivity, specificity, and predictive capacity, are described in the respective OECD validation documents and provide confidence in their use for the intended endpoints. In addition, there has been extensive work focused on individual chemicals to develop frameworks and guidance on how these assays could be integrated to assess the endpoints of interest (e.g., the Defined Approach for Skin Sensitization (OECD 2025a), the Integrated Approach on Testing and Assessment for Skin Corrosion and Irritation (OECD 2017), and the Guidance Document on Integrated Approach on Testing and Assessment (IATA) for Phototoxicity Testing (OECD 2024)). While the methods have been well evaluated for single chemicals, the performance of the selected assays for complex botanical mixtures remains an important area of investigation and is a central objective of the current work. Figure 1 illustrates how the selected assays address complementary dermal toxicity endpoints relevant to botanical safety assessment.

### In silico tools

*in silico* tools are increasingly applied to predict skin irritation and sensitization potential as part of integrated approaches to chemical safety assessment, especially for single chemicals (Johnson et al. 2020; Chilton et al. 2023). These computational models rely on chemical structure and mechanistic information to estimate the likelihood of adverse skin responses. The OECD Quantitative Structure–Activity Relationship (QSAR) Toolbox provides a freely available platform for grouping chemicals, identifying structural alerts, and filling data gaps through read-across and QSAR methods (Dimitrov et al. 2016). Commercial systems such as Derek Nexus (Lhasa Limited) use expert rule-based algorithms developed from published data on known sensitizers and irritants to provide categorical and numerical predictions (Langton 2019).

For botanicals, *in silico* tools need to be applied to individual chemicals on a constituent-by-constituent basis, and predictions can only be made for identified constituents with known structures. While *in silico* tools such as OECD QSAR Toolbox and Derek Nexus are limited to the evaluation of single chemicals with defined chemical structures, and therefore cannot account for unknown constituents or additive, antagonistic, or synergistic interactions within botanicals. The resulting data can be paired with NAMs data and weight-of-evidence evaluations to support regulatory submissions and safety assessments, or for screening to identify constituents of concern (Avonto et al. 2018).

For botanicals, *in silico* approaches are most appropriately viewed as screening tools that can identify known constituents with structural alerts for irritation, sensitization, or phototoxicity. However, because these approaches do not account for unknown constituents or mixture interactions, they are not intended to replace testing of the complete botanical preparation.

As part of this effort, *in silico* tool alerts on constituents will be compared with the assays with the whole mixtures and with the expected outcomes to evaluate their use.

### Skin irritation

**Figure 2.**
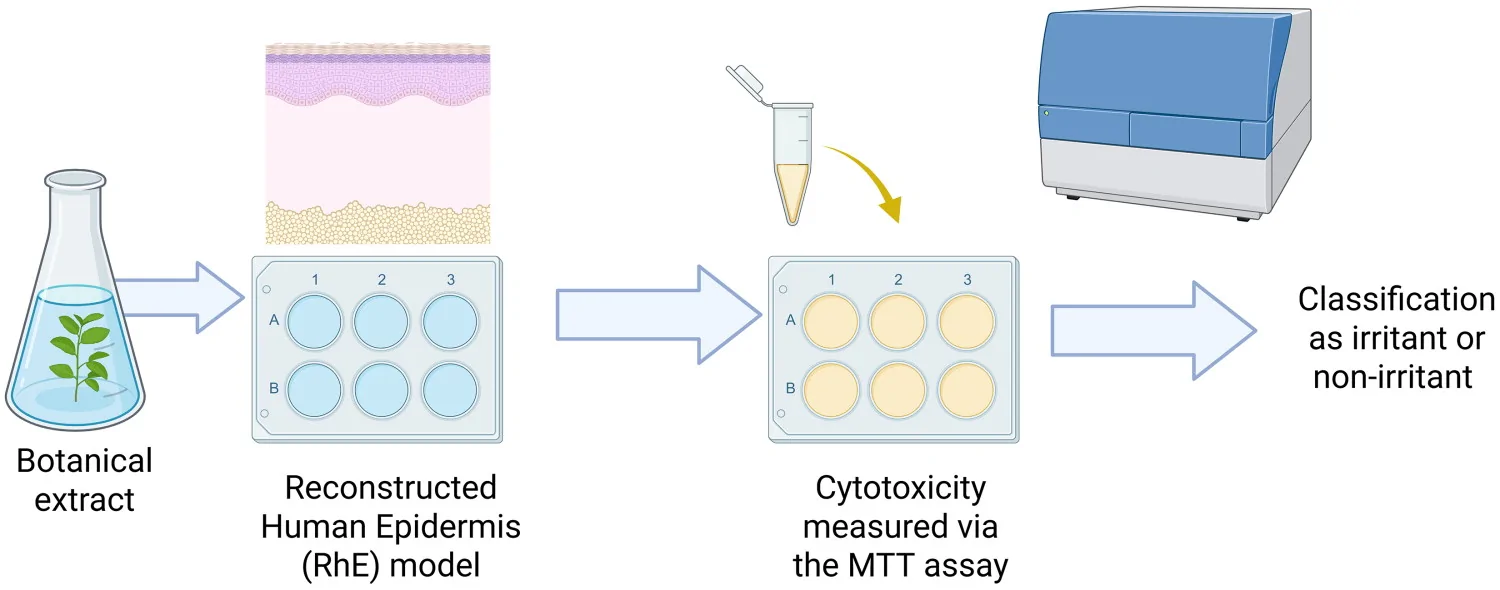
Simplified schematic of the dermal irritation assay using the reconstructed human epidermis (RhE) model; OECD test guideline 439 Skin Irritation test. Figure created in BioRender.

The OECD TG 439 *In vitro* Irritation: Reconstructed Human EpiDermis Test method (OECD 2025b), also referred to as the Skin Irritation Test (SIT) (Figure 2), utilizes the reconstructed human epidermis (RhE) model. This system replicates key structural and functional properties of the human epidermis. Test materials are topically applied for a prescribed exposure time, followed by a post-treatment incubation time. Tissue viability is then measured using the MTT (3-(4,5-dimethylthiazol-2-yl)-2,5-diphenyltetrazolium bromide) viability assay, with a mean viability of ≤50% serving as the classification threshold for irritation. The method does not distinguish between GHS Category 2 (irritant) and GHS Category 3 (mild irritant), nor does it identify corrosive substances (GHS Category 1), which are evaluated using OECD TG 431 method (OECD 2025d).

Botanicals may pose technical challenges with this test method, including direct reduction of MTT by botanical constituents and potential interference with MTT absorbance caused by pigments. However, the test method incorporates killed tissue control and colorant tissue control, and additional calculations can be performed to account for the potential direct reduction or assay interference. Solubility is not a limitation since materials are typically tested neat.

Botanicals are typically expected to have low levels of irritation and might be well-suited for other testing strategies like time-to-toxicity benchmark ranking approach (Alépée et al. 2022) or incorporation of mechanistic endpoints such as gene expression or cytokine profiling to offer complementary insights into the kinetics of tissue response and improve assay sensitivity and biological relevance for natural products.

However, the Working Group initially focused on regulatory-based assays, with such additional assays potentially being evaluated in future phases.

### Phototoxicity

**Figure 3.**
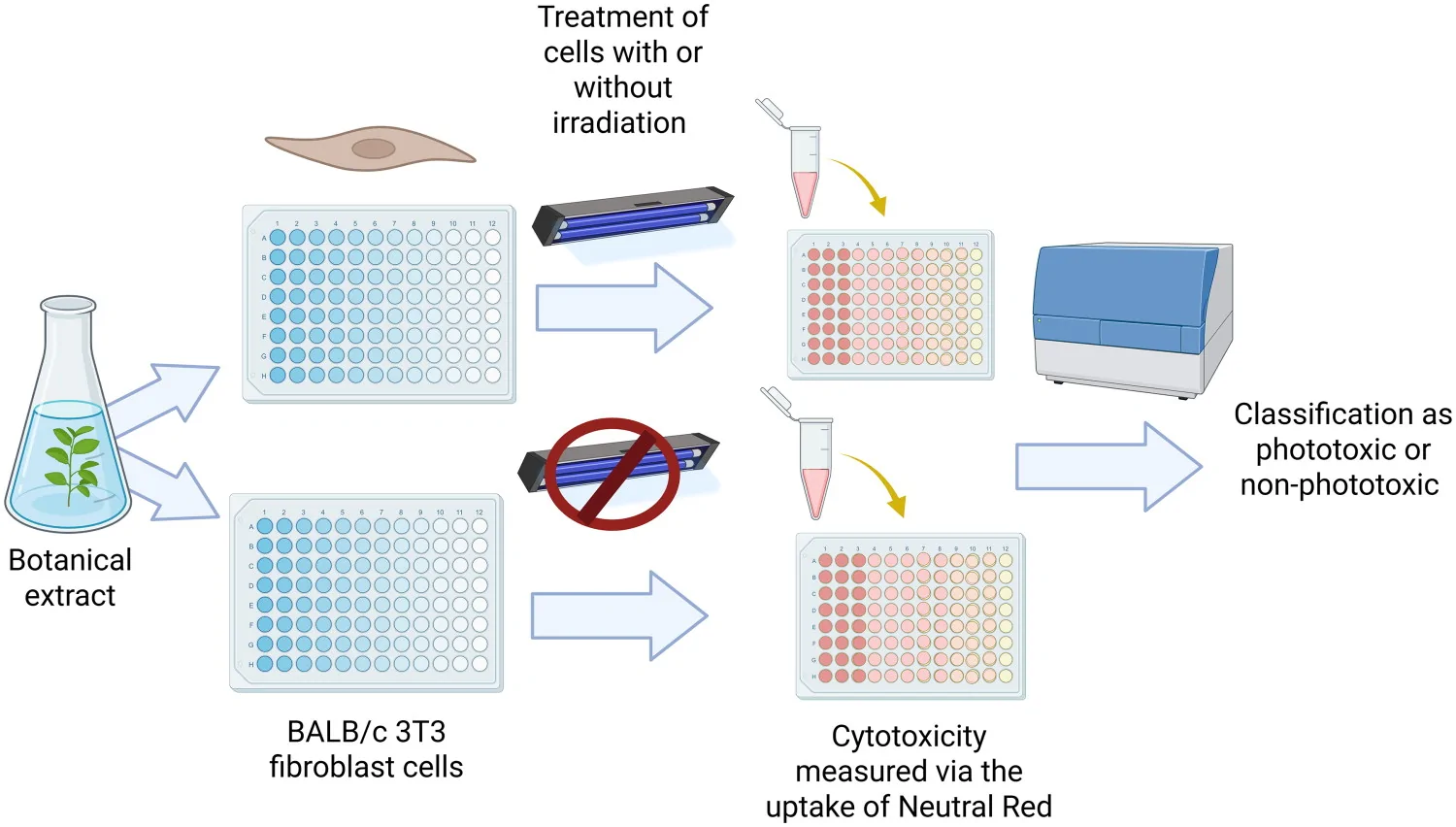
Simplified schematic of the dermal phototoxicity assay using the OECD TG 432 3T3 Neutral Red Uptake Phototoxicity Test. Figure created in BioRender.

The OECD TG 432, also known as the 3T3 Neutral Red Uptake (NRU) Phototoxicity Test, is a cell-based test method used to assess the phototoxicity (*i.e.,* photoirritation) potential of test compounds (Figure 3) (OECD 2019). A subconfluent monolayer of Balb/c 3T3 mouse fibroblast cells is exposed to at least 8 concentrations of the test compound in the presence and absence of ultraviolet A (UVA) and visible light (*i.e.,* irradiation). Cell viability is measured by neutral red uptake, and phototoxicity is evaluated using two metrics: the Photo Irritancy Factor (PIF) and the Mean Photo Effect (MPE). The PIF value compares the concentration of inhibition of neutral red at 50% (*i.e.,* inhibition concentration: IC_50_) in the presence and absence of irradiation. The MPE compares the dose response curves in the presence and absence of irradiation. Generally, a dose-range-finding trial is performed to select concentrations for at least 2 subsequent trials with a narrower dose range. Test compounds that are negative in this test method generally do not require further evaluation, and a positive or equivocal result is a flag for follow-up assessment (ICH 2015) (OECD 2024).

As a dilution-based assay, solubility is a common limitation of this test system; thus, given the complexity of botanicals, it may be a limitation for their evaluation in this system. Test compounds may be prepared in buffered salt solutions (*e.g.,* Hanks’ Balanced Salt Solution), dimethyl sulfoxide (DMSO), or ethanol; however, other solvents, as investigated by Cantrell et al. (2024), could be considered. And while complete solubility is desirable, useful comparisons between irradiated and non-irradiated cultures can still be made for test compounds with limited solubility up to the maximum tested concentration, as shown in Hilberer et al. (2025).

Additional considerations for testing botanicals within the applicability domain of the test method include whether the test method is applicable to compounds with UV absorption profiles predominantly or exclusively in the ultraviolet B (UVB) range, as these wavelengths are more toxic to cells. Because OECD TG 432 relies on UVA and visible light exposure, its applicability to botanicals containing constituents that absorb predominantly in the UVB range may be limited. Negative findings should therefore be interpreted cautiously when evaluating botanicals with known or suspected UVB-mediated photobiological activity. Despite these limitations, Hilberer et al. (2025) demonstrated this test method to be a high-throughput, cost-effective, and useful screening approach for identifying phototoxic botanicals.

OECD 432 (OECD 2019) was selected for testing the phototoxicity potential by the Working Group as this test method is often one of the first assays prescribed for phototoxicity evaluation (ICH 2015) and it was in alignment with the *in vitro* assay selected to evaluate 38 botanical ingredients intended as cosmetic ingredients (Hilberer et al. 2025). Future work can include additional, more complex test methods, such as the OECD TG 498 Reconstructed Human Epidermis Phototoxicity test method (OECD 2023). This TG may overcome solubility limitations, can incorporate UVB and UVA exposures, and has been used in tiered testing approaches to establish No Observed Effect Levels (NOELs) for phototoxicity (Ritacco et al. 2022; Hilberer et al. 2024). Photoallergy, which involves an immune-mediated response to light-activated compounds, is an important but distinct endpoint from phototoxicity. It is not a current focus of this manuscript but could be a future area of interest for the Working Group.

### Skin sensitization

OECD Guideline (GL) 497 (OECD 2025a) describes defined approaches for assessing skin sensitization using NAMs. These approaches integrate data from multiple assays, such as the DPRA, KeratinoSens^™^, and h-CLAT, along with computational models and decision logic to predict sensitization hazard and potency. Each test method corresponds to defined key events (KE) in the Adverse Outcome Pathway (AOP) for skin sensitization (OECD 2014), including protein reactivity (KE1), keratinocyte activation (KE2), and dendritic cell maturation (KE3). Each of the test methods provides a binary hazard classification of skin sensitization potential, and together in combination with *in silico* data, additional information on potency may be determined (OECD 2025a). The final classification of a test substance is based on concordant results from two of three key event assays, referred to as the ‘2 out of 3’ defined approach (OECD 2025a).

For the purposes of this effort, the OECD Guideline 497 ‘2 out of 3’ defined approach will initially be considered for botanical mixtures in the same manner as it is applied to single chemicals. However, because these approaches were primarily developed and validated using single chemicals, the extent to which concordant results across key events support hazard identification for complex botanical mixtures remains an important area of investigation. As such, the suitability of this approach will be an area for evaluation in future work.

#### Direct Peptide Reactivity Assay (DPRA)

**Figure 4.**
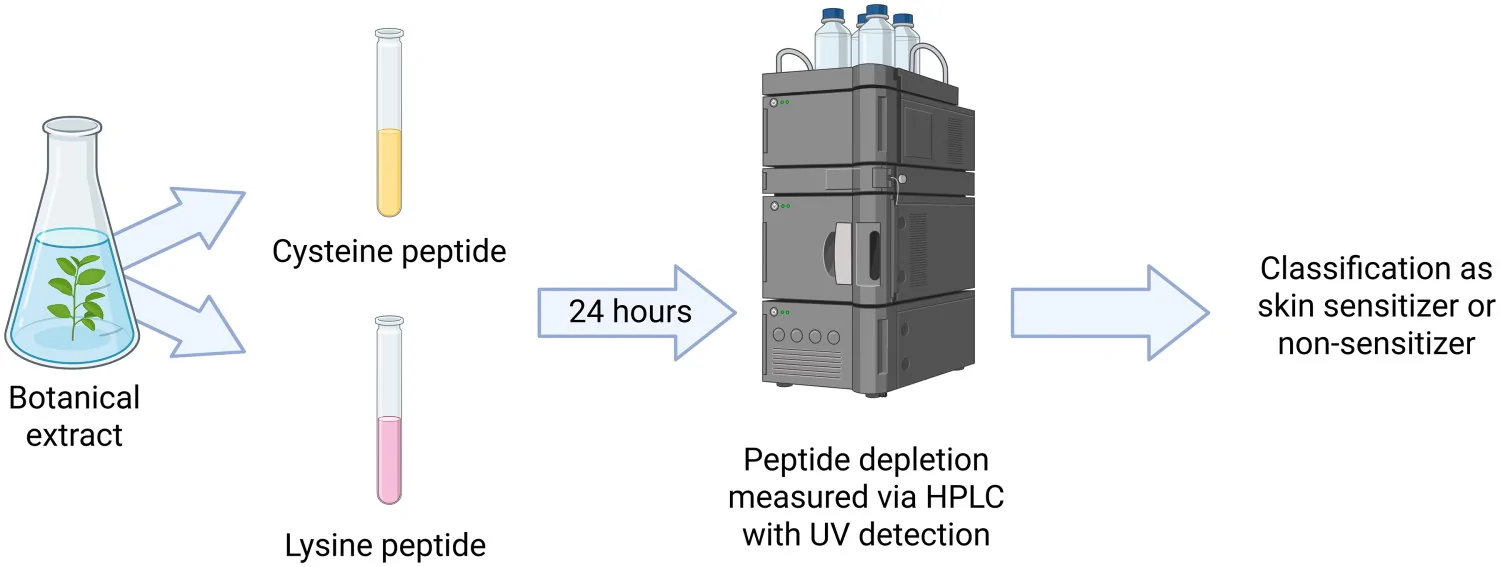
Simplified schematic of the Direct Peptide Reactivity Assay (DPRA) test system; OECD test guideline 442C *in chemico* skin sensitization assay addressing the adverse outcome pathway key event on covalent binding to proteins. Figure created in BioRender.

The Direct Peptide Reactivity Assay (DPRA) is an *in chemico* assay included in OECD TG 442C covering Key Event 1 (KE1) in the AOP (OECD 2014, 2025a, 2025c) and evaluates chemical haptenization of proteins (Figure 4). Test substances are prepared at a stock concentration and then incubated with a cysteine- and a lysine-containing peptide in two separate experiments. The interactions of the test compound with the peptides are measured by high performance liquid chromatography analysis. Test compounds that have skin sensitization potential bind to the peptides (cysteine and lysine), resulting in their depletion from the reaction mixtures.

Botanicals are complex mixtures without defined molecular weights and, therefore, cannot be prepared at the prescribed 100 mM stock concentration. However, gravimetric approaches estimating molecular weights are considered (OECD TG 442 C; OECD 2025c), and have been specifically investigated for plant extracts (Kolle et al. 2023) with preliminary success. The DPRA offers the advantage that several solvents, including organic solvents, can be used to prepare the test compound; however, the mixture must also be fully soluble and not precipitate when added to the aqueous peptide reaction mixture. Given their complexity, full solubility of botanicals in both stock and reaction mixtures is needed for a definitive assessment, particularly when there is no reactivity with cysteine- or lysine-containing peptides. An additional consideration is the nonspecific binding of tannins and certain other phenolic compounds, commonly found in plants, to peptides. A final consideration is the potential for co-elution, as constituents in the extract may have the same retention time as the cysteine- or lysine-containing peptides. Co-elution controls are included in the assay and serve as a check for potential HPLC-detection interference.

#### Activation of Keap1-Nrf2-ARE

**Figure 5.**
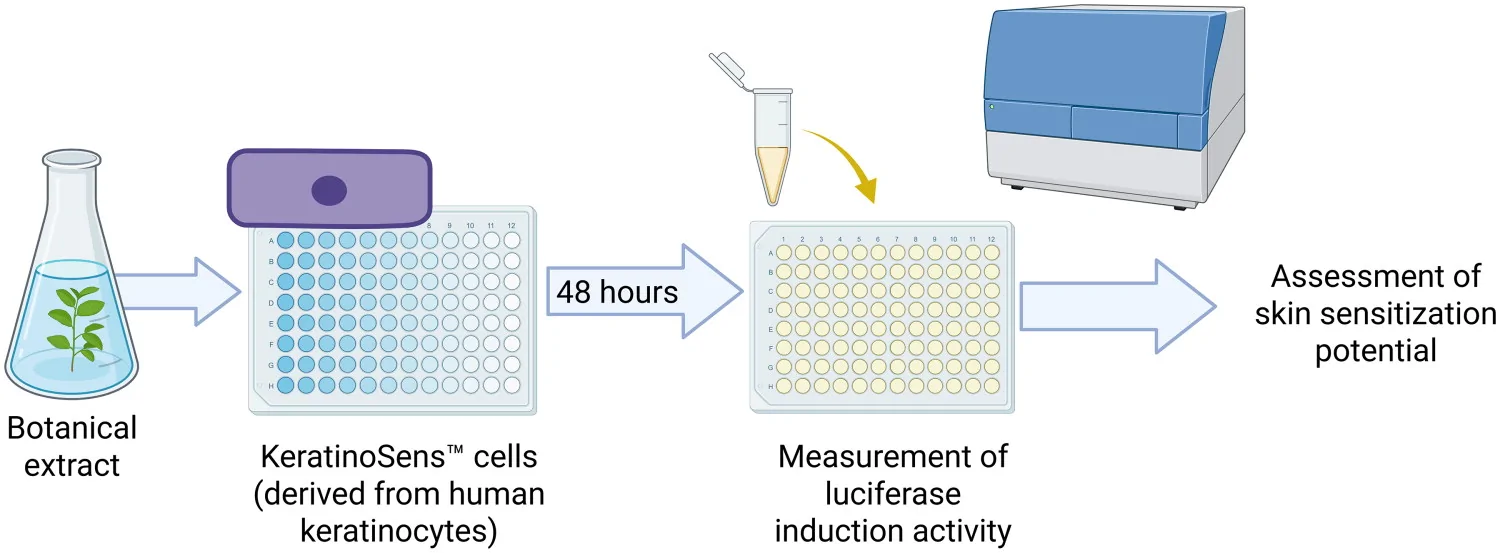
Simplified schematic of the KeratinoSens^™^ test method measuring the activation of keratinocytes *via* the Keap1-Nrf2-Antioxidant Response Element (ARE) pathway; OECD Test Guideline 442D in vitro skin sensitization assays addressing the Adverse Outcome Pathway Key Event on keratinocyte activation. Figure created in BioRender.

The KeratinoSens^™^ is a cell-based reporter gene assay included in OECD TG 442D covering Key Event 2 (KE2) in the AOP (OECD 2024a) to evaluate chemical-induced keratinocyte activation *via* the Keap1-Nrf2-Antioxidant Response Element (ARE)-dependent pathway (Figure 5). The test system uses an immortalized HaCaT human keratinocyte cell line transfected with a plasmid containing the luciferase gene. Interaction of sensitizers with the Keap1 protein results in dissociation of the Nrf2 transcription factor, and subsequent transcription of ARE-dependent genes, including luciferase. Luciferase induction and viability (*via* MTT assay) are measured for the test compound at 12 concentrations. A 1.5-fold luciferase induction relative to the respective solvent control and relative viability are used to evaluate activation of the keratinocytes. A test compound is predicted to activate skin keratinocytes if cellular viability is >70%, the luciferase induction is statistically significant and ≥1.5-fold as compared to the solvent/vehicle control, and there is a concentration -dependent increase in luciferase induction.

Botanicals may pose challenges for this test system due to solubility, potential interference with the MTT endpoint, and the risk that cytotoxic components may mask responses induced by sensitizing constituents. Further, as outlined in OECD TG 442D (2024a), phytoestrogens at high concentrations can interfere with the luciferase enzyme. Regarding solubility, a final preparation into aqueous culture medium containing 1% DMSO is necessary to support the cell monolayer over the 48-hour treatment exposure period. Complex mixtures, which often pose solubility challenges, have been evaluated in the KeratinoSens^™^ assay (Andres et al. 2013; Puginier et al. 2022; Strickland et al. 2022; Islas-Robles et al. 2025). While full solubility is ideal, homogenous suspensions may be evaluated if bioavailability can be confirmed (*e.g.,* dose-dependent cytotoxicity response). Test compounds that may directly reduce MTT or are dark-colored, as described above in OECD TG 439 (OECD 2025b), may interfere with the MTT endpoint; however, the treatments are removed from the cell cultures prior to measurement of this endpoint, reducing the likelihood of interference. If interference is observed, additional viability endpoints, such as neutral red uptake or Prestoblue^™^, may be considered.

#### Human cell line activation test (h-CLAT)

**Figure 6.**
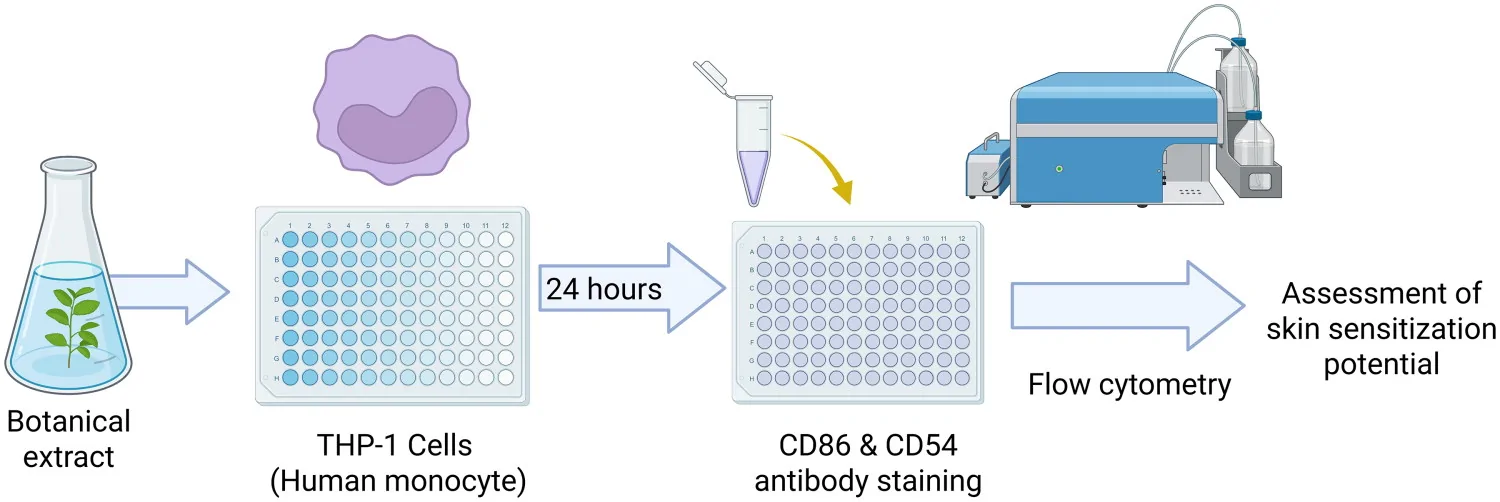
Simplified schematic of the human cell line activation test (h-CLAT) test method measuring the chemical-induced activation of dendritic cells using the immortalized human monocytic leukemia cell line, THP-1; OECD Test Guideline 442E *In vitro* skin sensitisation assays addressing the Key Event on activation of dendritic cells on the Adverse Outcome Pathway for skin sensitisation. Figure created in BioRender.

The h-CLAT is a cell-based assay included in OECD TG 442E that is used to address Key Event 3 (KE3) in the AOP (OECD 2014; OECD 2024b; OECD 2025a) through chemical-induced activation of dendritic cells using the immortalized human monocytic leukemia cell line, THP-1, as a surrogate for dendritic cells (Figure 6). THP-1 cells mature and increase expression of specific cell-surface markers, including CD86 and CD54, in the presence of a skin sensitizer. In the h-CLAT assay, THP-1 cells are exposed to 8 concentrations of the test material for 24 h, then stained with fluorescent antibodies against CD86 and CD54, and analyzed by flow cytometry. Cell viability is measured concurrently in the same cell population using propidium iodide staining. Increases in the relative fluorescent intensity ≥150% for CD86 and/or ≥ 200% for CD54 are indicative of dendritic cell activation. A dose-range-finding cytotoxicity trial is performed initially to inform doses for subsequent trials, and two concordant results from independent experiments are required for assessment.

As with the KeratinoSens^™^ method, there are similar considerations for testing botanicals in the h-CLAT, specifically solubility and the possibility that cytotoxic constituents may mask responses to sensitizing ones. The three solvents used to prepare test materials in the h-CLAT include DMSO, culture medium, and saline, and while other solvents might be suitable, they require further investigation prior to use (Islas-Robles et al. 2025). The test method is applicable to test materials that are soluble or form a homogenous suspension, but test materials with Log Kow >3.5 may produce false negatives (OECD 2024b). While the test method is applicable to various types of materials, few studies have presented its use to assess botanicals and yielded mixed results. In Kolle et al. (2023), botanical extracts induced expression of CD54 (5 of which were non-sensitizers *in vivo*), whereas Shiraishi et al. (2021) reported Brazilian green propolis (a resinous mixture sourced mainly from *Baccharis dracunculifolia*) to be negative for skin sensitization in the h-CLAT but a moderate sensitizer in the local lymph node assay.

For skin sensitization potential, the three skin sensitization test methods selected were the first adopted by the OECD. These test methods have been used to evaluate more complex materials, including botanicals, and can be adapted to evaluate non-discrete materials without defined molecular weights (Andres et al. 2013; Kolle et al. 2023; Islas-Robles et al. 2025). There are other most recently adopted methods, such as GARDskin, that can be used to measure KE3 (Johansson et al. 2019), or the EpiSensA assay for KE2 (Saito et al. 2017). These additional assays have demonstrated greater compatibility with difficult-to-test substances and may be considered by the Working Group following this first phase of work.

## Botanicals

Eleven botanicals were selected by the BSC Dermal Working Group for evaluation of the suitability of *in vitro* test methods for dermal toxicity assessment (Table 1). Selection criteria included the availability of supporting information from human clinical case reports or studies, *in vivo* animal studies, or mechanistic data from *in vitro* models. Botanicals were selected to represent a range of expected outcomes in the assays, including those anticipated to induce dermal irritation, sensitization, or phototoxicity, as well as those not expected to induce any of the adverse outcomes described above. Preference was given to materials with at least partial chemical characterization (*e.g.,* quantification of key constituents) and extract types commonly used in personal care products, such as ethanol extracts, essential oils, or CO_2_ extracts. In some instances, botanicals not used in consumer products but with well-documented dermal effects (*e.g.,* poison ivy, cypress spurge [*Euphorbia cyparissias* L., Euphorbiaceae]) were included to evaluate assay performance. All materials were sourced from reputable suppliers and have undergone or will undergo chemical verification consistent with prior BSC work (Waidyanatha et al. 2024). For bergamot essential oil, one lot was produced by cold pressing and therefore contains furanocoumarins. A second lot was further processed by steam distillation, which facilitates the removal of less-volatile compounds, including furanocoumarins.

**Table 1.** Candidate botanicals selected for future characterization and testing.

Standardized common name	Scientific name	Plant part	Extract type	Anticipated dermal assay response	Evidence types and key references
Asian ginseng	*Panax ginseng* C.A Mey	Root	95% ethanol	None	Humans (Becker et al. 2015) Mice (Shin et al. 2005; Bae et al. 2006; Shin et al. 2006; Kim et al. 2008; Kim et al. 2011) *in vitro* (Lee et al. 2007; H.-S Lee et al. 2012)
Cinnamon	*Cinnamomum zeylanicum* J.Presl	Bark	Essential oil (steam distilled)	Irritation/ sensitization	Humans (Calnan 1976; Sparks 1985; Nixon 1995; Lauriola et al. 2010; Connolly et al. 2017) Rabbits (Bickers et al. 2005).
Cypress spurge	*Euphorbia cyparissias* L.	Mixed plant parts	95% ethanol	Irritation	Humans (Gminski et al. 1998, Fleischman et al. 2012) Animals (Cristina et al. 2014)
Goldenseal	*Hydrastis canadensis* L.	Root & rhizome	95% ethanol	Unknown	
Hops	*Humulus lupulus* L.	Cones	CO_2_	None	Humans (Almaguer et al. 2014, Becker et al. 2024, Hurth et al. 2022)
Lavender	*Lavandula angustifolia* Mill.	Flowering tops	Essential oil (steam distilled)	None	Humans (Opdyke 1979; Mekonnen et al. 2019) Animals (Forbes et al. 1977; Opdyke 1979)
Bergamot	*Citrus × limon* (L.) Osbeck, syn. *C × bergamia* (Risso) Risso & Poit.	Fruit peel	Essential oil (cold-pressed)	Phototoxicity	Humans (Zaynoun et al. 1977; Girard et al. 1979; Gardner and McGuffin 2013) Animals (Yasui and Hirone 1994, Young et al. 1990) *in vitro* (Ashwood-Smith et al. 1980; Gardner and McGuffin 2013)
Furanocoumarin-free Bergamot	*Citrus × limon* (L.) Osbeck, syn. *C × bergamia* (Risso) Risso & Poit.	Fruit peel	Essential oil (cold-pressed then steam-distilled)	None	*in vitro* (Kejlová et al. 2007)
Parsley	*Petroselinum crispum* (Mill.) Fuss	Seed	CO_2_	Phototoxicity/ irritation/ sensitization	Humans (Kauppinen et al. 1980; Rademaker 1999; Janusz and Schwartz 2021, Bellringer 1949) Animals (Griffiths and Douglas 2000)
Poison ivy	*Toxicodendron radicans* (L.) Kuntze	Mixed plant parts	95% ethanol	Sensitization	Humans and animals (McNair 1923; Simon 1936) Animals (Kim et al. 2016; Liu et al. 2019; Rashid et al. 2019) *in vitro* (Gao et al. 2024)
Thyme	*Thymus zygis* L.	Aerial parts, including flower & leaf	Essential oil (steam distilled)	Irritation/ sensitization	Humans (Spiewak et al. 2001) Animals (Rojas-Armas et al. 2019; Uter et al. 2010; Sindle and Martin 2021) *in vitro* (Xu et al. 2006)

Below, we provide information on each botanical regarding dermal toxicity. The literature supporting botanical selection was compiled through a nonsystematic review of publicly available sources (e.g., PubMed, Google Scholar) using botanical names in combination with endpoint-relevant search terms. Only evaluations of the same study species, or specific constituents identified by the expert group as likely being in the selected extract, were included in the review. Representative endpoint search terms included irritation, sensitization, allergy, dermal, skin permeation enhancement, local tolerance, dermatitis, photoreactive, patch test, phototoxicity, hypersensitivity, adverse reactions, immunogenicity, transdermal absorption, erythema, eschar, edema, Draize, guinea pig, open epicutaneous test, Buehler test, Freund’s complete adjuvant test, optimization test, split adjuvant test, guinea pig maximization test, and relevant OECD Test Guidelines and *in vitro* methods. This list is not exhaustive and was supplemented by additional terms identified through expert input from the Working Group and review of the literature. Human, animal, *in vitro*, and authoritative review sources were considered when available. Studies including some degree of phytochemical characterization of the study agent were prioritized, recognizing that detailed compositional information is not consistently provided in the published literature. Consistent with previous Botanical Safety Consortium publications focused on supporting study design and botanical selection, the literature was not collected or evaluated using a formal systematic review methodology, and study quality was not formally assessed (Patel et al. 2023). The determination of the composition and concentrations of selected marker compounds for each of the test materials is underway as part of the initiative, and results of the chemical analysis will be shared together with the bioassay test results.

### Asian ginseng

Asian ginseng (*Panax ginseng*, Araliaceae) is a perennial herb native to the mountainous regions of China and Korea. It has been cultivated for centuries. Asian ginseng is reported to improve vitality, increase stamina, and promote overall health (WHO 1999a; EMA 2024; Kim et al. 2024). The main benefits of Asian ginseng have been linked to its content in dammarane-type saponins, called ginsenosides, but the root also contains polysaccharides, polyacetylenes, and phenolic acids (Jee et al. 2014; Ru et al. 2015).

Two human studies used 48-hour occlusive patch tests with Asian ginseng root extract in petroleum jelly. Both studies reported no visible reactions or adverse effects at 24 and 48 h after removal (Unpublished data submitted by the Personal Care Products Council and cited by Becker et al. 2015).

Several studies examined the dermal or skin-care benefits of Asian ginseng in mice. None reported sensitization or irritation following topical administration (Shin et al. 2005; Bae et al. 2006; Shin et al. 2006; Kim et al. 2008, 2011).

*In vitro* investigations of fermented red ginseng (*P. ginseng* steamed 98 °C–100 °C for 2–3 h) and white ginseng (air-dried *P. ginseng*) extracts suggest that these substances do not cause irritation or sensitization of the epidermis (Lee et al. 2007, 2012).

The U.S. National Center for Complementary and Integrative Health (NCCIH) notes that there is insufficient evidence to support Asian ginseng’s safety topically (NCCIH 2025a). However, this is based on a lack of safety studies, not on evidence of toxicity. Additionally, they reported that short-term oral use is generally considered safe (up to 6 months). Numerous studies evaluated Asian ginseng root in humans and animals following oral administration, and *in vitro* systems have not reported toxicity (National Toxicology Program 2011; N.-H. Lee et al. 2012).

Based on current evidence, Asian ginseng has not been associated with dermal toxicity; therefore negative results are expected in the evaluated NAMs.

### Cinnamon

Cinnamon bark (*Cinnamomum zeylanicum*, Lauraceae*)* is derived from an evergreen tree native to Sri Lanka. The dried inner bark has long been used as a spice and is also used in traditional medicine, particularly for gastrointestinal conditions and other indications (NCCIH 2024). Cinnamon essential oil is produced by steam distillation of the bark. It contains high levels of cinnamaldehyde [DSSTox substance identifier (DTXSID)1024835], the compound responsible for its warm, spicy aroma. Other important constituents are eugenol [DTXSID9020617], cinnamyl acetate [DTXSID2044765], and linalool [DTXSID7025502] (Guo et al. 2024). Commercially, cinnamon bark oil is used as a flavoring in food, a fragrance in cosmetics, and an active component in aromatherapy. This botanical was selected for its ability to act as a skin irritant and sensitizer (WHO 1999b; Malaysian Government’s Medicinal Herbs & Plants Database 2025; Royal Botanic Gardens, Kew 2025).

Numerous case reports documented allergic contact dermatitis, chemical burns, and oral mucosal irritation with systemic symptoms following exposure to cinnamon-containing products (Calnan 1976; Sparks 1985; Nixon 1995; Lauriola et al. 2010; Connolly et al. 2017). Occupational allergic contact dermatitis has been documented among spice handlers and perfumery workers (Ackermann et al. 2009).

There is also evidence that some of the individual constituents of cinnamon essential oil can cause skin irritation and sensitization, especially cinnamaldehyde and cinnamyl alcohol [DTXSID9041491] (Bickers et al. 2005; European Commission 2020). The Research Institute of Fragrance Materials (RIFM) expert panel has an extensive review on cinnamyl alcohol, cinnamaldehyde, and cinnamic acid [DTXSID40110056] (Bickers et al. 2005). Overall, they concluded that cinnamaldehyde can cause skin irritation and sensitization, with irritation emerging at moderate concentrations and strong reactions at high or undiluted levels. Cinnamyl alcohol shows low irritation potential and only induces sensitization at relatively high concentrations, while cinnamic acid produces only slight irritation even at higher doses and does not sensitize the skin. Some of the individual studies included in the RIFM review are discussed below, but in a few cases, the cited RIFM studies are not available in the public domain.

A human repeated-insult patch test with 8% cinnamon oil in petrolatum, conducted on 28 participants, reported no irritation or sensitization (Marzulli and Maibach 1980). However, Bickers et al. (2005) reported that cinnamaldehyde caused dose-dependent irritation, with no reactions at ≤ 1.25% but severe effects at 8%. Other constituents, cinnamyl alcohol and cinnamic acid, remained nonirritant up to 10% and 4%, respectively.

In animal studies, the three constituents (cinnamyl alcohol, cinnamaldehyde, and cinnamic acid) exhibited variable irritant responses depending on dose and test conditions. Cinnamaldehyde caused moderate to severe erythema and edema in rabbits when applied undiluted, but no irritation at concentrations ≤ 5% (Bickers et al. 2005).

Several studies have demonstrated beneficial dermal effects of cinnamon extracts, including accelerated wound healing, enhanced re-epithelialization, increased antioxidant capacity, and stimulation of keratin biosynthesis (Farahpour and Habibi 2012; Daemi et al. 2019; Seyed Ahmadi et al. 2019). Cinnamon oil’s phototoxicity potential appears low, as the neat oil produced no effects in mouse and pig studies (Forbes et al. 1977). Some *in vitro* studies have described potential anti-aging properties of cinnamaldehyde and cinnamon extracts (Takasao et al. 2012; Kossyvaki et al. 2020).

Recent *in vitro* work further suggests that environmental UVB exposure can amplify cinnamaldehyde’s sensitization potential by promoting photo-oxidative activation (Patel et al. 2024).

Overall, cinnamon bark essential oil is expected to elicit positive responses in dermal irritation and sensitization tests.

### Cypress spurge

Cypress spurge *(Euphorbia cyparissias*, Euphorbiaceae) is a perennial flowering plant native to Europe and introduced to North America (New York State Department of Environmental Conservation 2025). When damaged, the stem and leaves exude milky-white latex. Medicinally, the root juice in vinegar has been used for constipation or as a diuretic, and the latex has been applied to treat warts and corns (Gminski et al. 1998). The latex of cypress spurge contains flavonols, diterpenoid esters, and triterpenoids, although information about the composition is limited. From a toxicological perspective, the diterpenoid esters are the most relevant compounds. However, only a few of them have been characterized (Ott and Hecker 1981), including cyparissins A and B from the aboveground parts (Lanzotti et al. 2015), and 12-O-tetradecanoylphorbol-13-acetate [DTXSID5023798] in the whole plant (Fiedler et al. 2023). Even for these, many compounds lack Chemical Abstracts Service Registry Numbers (CASRNs) or other identifiers. The latex from this plant is known to be a skin irritant (Gucker 2010). Topically, the plant’s latex, even after drying, can cause reddening, itching, burning, and blisters (Gminski et al. 1998).

It is difficult to find primary literature for cypress spurge relating to its ability to cause irritation in humans, though many government websites report its ability and cite review books like Westbrooks and Preacher (1986) and Stephens (1984). It has been reported that the latex was used to delay the healing of wounds to avoid being enrolled in the army (Gilca et al. 2018). A case study showed a woman developed keratouveitis (a serious eye condition involving inflammation of the cornea and the anterior uvea) after her hands, exposed to cypress spurge sap while she was gardening, were used to rinse her eyes (Fleischman et al. 2012).

One study explored cypress spurge’s use as an ointment on rats, dogs, and healthy human volunteers (Cristina et al. 2014). The response in rats ranged from ‘medium irritant’ to ‘strong irritant’, and histological examination of the skin showed worsening of contact dermatitis. At lower concentrations, dogs showed light cutaneous reactions. In humans, burning and irritation were reported.

*In vitro*, cypress spurge extracts inhibited multiple digestive and metabolic enzymes and showed dose-dependent cytotoxicity in human cell lines, with stronger effects from the ethanolic extract than the aqueous extract (Alonazi et al. 2020). Another *in vitro* study showed extracts inhibited P-glycoprotein (P-gp) activity (an ATP-dependent efflux transporter involved in limiting absorption and promoting excretion of xenobiotics) and caused concentration-dependent cytotoxicity and oxidative stress in human keratinocyte and dermal fibroblast cells, with the methanolic extract producing stronger effects compared to an aqueous preparation (Jadranin et al. 2023). A separate study in ovarian cell lines also showed that constituents from cypress spurge (cyparissins A and B) inhibit P-gp activity (Lanzotti et al. 2015).

Based on the human and animal data, cypress spurge is expected to induce dermal irritation in the selected assays.

### Goldenseal

Goldenseal (*Hydrastis canadensis*, Ranunculaceae) is a perennial, herbaceous flowering plant, and is native to the eastern regions of the United States and Canada. Its root is purported to have antimicrobial and anti-inflammatory effects and has traditionally been used to treat various health conditions, including skin problems, digestive issues, and urinary tract infections (NCCIH 2025b; Roe et al. 2025). The roots contain isoquinoline alkaloids, notably berberine [DTXSID9043857] and hydrastine [DTXSID9025409], with smaller amounts of canadine [DTXSID5022724] and other alkaloids (Bradley 1992; Le et al. 2014). This botanical was previously selected by other Working Groups for its potential to induce botanical-drug interactions (Roe et al. 2025). It was nominated for this group based on evidence that berberine may cause skin irritation.

Berberine is a naturally occurring alkaloid that has been reported as having photosensitizing properties, causing increased *in vitro* cytotoxicity when exposed to UV (Jantová et al. 2006) and has been demonstrated as being phototoxic to mosquitoes co-exposed to berberine and UV (Philogène et al. 1984). One study reported that berberine can ease the effects of dermatitis in mice when administered orally (Andoh et al. 2021). Chen et al. examined chemical-induced dermatitis in mice and reported that berberine could reduce its effects. However, in mice treated only with berberine alone, skin irritation occurred at 2 of the 4 doses (2024). Another study investigated the effects of dermal exposure to *Berberis aristata* (which contains berberine) formulations on mice and reported that it could alleviate psoriasis-like symptoms, but it did not test *B. aristata* alone (Shrivastav et al. 2022).

In a mouse skin carcinogenicity study, topical application of berberine sulfate [DTXSID70977452] reduced both the number of tumors and the percentage of animals developing tumors in a standard initiation-promotion model (Nishino et al. 1986). Mice were first exposed to a carcinogen and then repeatedly exposed to a tumor promoter, with berberine sulfate applied topically before each promotion treatment for a subset of the mice. Animals receiving the berberine sulfate treatment developed fewer tumors than untreated controls, demonstrating a strong inhibitory effect. The dermal effects of berberine sulfate alone were not evaluated.

Overall, while there is some evidence that berberine may be an irritant, there is insufficient evidence to conclude that goldenseal is an irritant. As such, the anticipated effects of the goldenseal extract in the selected assays are unknown. This botanical case study is still valuable as an example in a ‘gray’ area of expected response and to help understand differences between strong positives and negatives.

### Hops

Hops (*Humulus lupulus*, Cannabaceae) are a climbing perennial vine native to Europe, western Asia, and North America, and have been introduced as cultivars to temperate regions of the world (Chadwick et al. 2006; National Institute of Diabetes and Digestive and Kidney Diseases 2012). Hops cones and flowers are primarily used as flavoring agents in beer production. Hops are also used as a purported antimicrobial and antiperspirant agent, as well as a fragrance ingredient in deodorants, creams, and lotions (Almaguer et al. 2014; Becker et al. 2024). The major constituents in the CO_2_ extract used in the dermal tests are bitter principles, which are prenylated polyketides that include humulone [DTXSID50946227], cohumulone [DTXSID701318500], adhumulone [DTXSID901359439], as well as lupulone [DTXSID00875553], colupulone [DTXSID80963664], and adlupulone [DTXSID901318318] (Chadwick et al. 2006; WHO 2007; Becker et al. 2024). Hops were selected as a negative case study for dermal toxicity (Becker et al. 2024). Some CO_2_ extracts also contain prenylated flavonoids such as 8-prenylnaringenin [DTXSID60333083], which is one of the most potent phytoestrogens (Milligan et al. 2002; Štulíková et al. 2018).

Although hops were selected for their expected lack of effects, allergic reactions have been reported among hop harvesters and brewery workers regularly exposed to fresh or dried hops (Chadwick et al. 2006; Becker et al. 2024). Follow-up work, including skin prick tests in farmers, suggested that type I allergy to hops is less likely, with contact dermatitis being the most probable cause (Becker et al. 2024). Contact dermatitis has been attributed to myrcene [DTXSID6025692] present in fresh hop oil, with positive patch test reactions observed for the fresh oil but negative results for dried, crushed flowerheads (Almaguer et al. 2014; Becker et al. 2024). Mechanical dermatosis has also been associated with abrasion from rough stem hairs and secretions of lupin from the yellow glandular hairs on the scales of the strobiles (WHO 2007; Almaguer et al. 2014). Allergic reactions appear to occur only after external contact with the herb or oil, and are not likely when using hop extract, since allergens are believed to be removed during extraction (Almaguer et al. 2014).

Human irritation and sensitization studies conducted with hops extract have consistently demonstrated no evidence of irritation or sensitization. Formulations containing hop extract were negative in a 2-week cumulative irritation test at 0.125%, as well as in patch tests at 0.06–0.12% and 0.18% (Becker et al. 2024). Similarly, no sensitizing effects were observed in human sensitization assays, including a human maximization test using 0.125% extract and several human repeat insult patch tests using 10% extract in butylene glycol, 0.18% in caprylic/capric triglyceride, and 5% in a glycerin/water vehicle (Becker et al. 2024).

A randomized, prospective, placebo-controlled, double-blind UVB erythema study involving 40 healthy volunteers evaluated the skin tolerability of a cream containing 1% hops extract (Hurth et al. 2022). Patch testing on non-irradiated skin showed that the cream with hops extract was well tolerated, with no increase in erythema index compared with the placebo formulation, while patch testing on UVB-irradiated skin showed that the formulation with hops extract had a statistically significant reduction in erythema index compared with the placebo formulation (Hurth et al. 2022).

Based on these results, the selected hop extract is not expected to elicit positive effects in the selected assays.

### Lavender

Lavender (*Lavandula angustifolia*, Lamiaceae) is a perennial aromatic shrub native to the Mediterranean region, particularly cultivated in southern Europe (Castle and Lis-Balchin 2002). It is widely used for its purported calming and soothing properties, with essential oil and flower preparations employed in aromatherapy, herbal medicine, and cosmetics. The plant’s flowers contain phenolic compounds such as rosmarinic acid [DTXSID20896987] and glycosides of the flavonoids apigenin [DTXSID6022391] and luteolin [DTXSID4074988] (Zhao et al. 2015; Héral et al. 2021; Mykhailenko et al. 2025). Compounds associated with the aromatic properties of the essential oil include linalool acetate [also known as linalyl acetate; DTXSID7026946], linalool, lavandulyl acetate [DTXSID30904865], and other terpenes (Wilson et al. 2021).

Based on animal and human studies, the irritation potential of lavender essential oil appears to be low to mild, and it is species- and preparation-dependent. Undiluted oil was nonirritating on hairless mice and swine, while slight irritation occurred on occluded rabbit skin; a 10% ointment caused no irritation in rabbits, and 16% in petrolatum produced no irritation in a 48-hour human closed-patch test (Opdyke 1979; Mekonnen et al. 2019).

The sensitization potential has been shown to be generally low in human studies: a 16% maximization test (*i.e.*, an assay exposing the skin to very harsh conditions in order to assess the tolerability of an ingredient) in 25 volunteers elicited no sensitization (Opdyke 1979). However, sensitization has been reported with oxidized essential oil and with oxidized linalool and linalyl acetate, suggesting freshness and proper storage are important (Hagvall and Christensson 2016; Uter 2017). Phototoxicity has not been observed. Neat lavender essential oil was not phototoxic in mice and pigs (Forbes et al. 1977; Opdyke 1979).

It is worth noting that there is an ongoing debate over the ability of lavender essential oil (and other essential oils) to disrupt endocrine activity (Ramsey et al. 2019). The National Institute of Environmental Health Sciences (NIEHS) tested lavender essential oils and several of their constituents *in vitro*, showing changes in hormone levels (Henley et al. 2007), but noted a need for epidemiological studies to confirm these findings. There are several reviews and research papers, many of which conclude that the evidence is lacking (Hawkins et al. 2020; Hawkins et al. 2022). A full exploration of all the evidence and a judgment is beyond the scope of this article. However, the potential presence of phytoestrogens in the essential oil may impact the results of the KeratinoSens^™^ assay, as previously discussed.

Overall, lavender essential oil is expected to show low irritation and sensitization potential, and a lack in phototoxicity. As such, the lavender essential oil is not expected to elicit effects in the selected assays.

### Bergamot

Bergamot orange (*Citrus × limon*, syn. *C × bergamia*, Rutaceae) is a hybrid of bitter or sour orange with lemon or lime, cultivated in Italy, Africa, the Americas, and China (Navarra et al. 2015; Ferlazzo et al. 2016; Valussi et al. 2021). Its peel contains essential oil and other bioactive compounds used in cosmetics, perfumes, and pharmaceuticals, as well as for flavoring, most notably as part of the flavor of Earl Grey tea (Navarra et al. 2015; Ferlazzo et al. 2016). In Italian folk medicine, bergamot preparations have been used for fever, parasitic diseases, wound healing, and infections of the mouth, skin, and respiratory and urinary tracts (Navarra et al. 2015). The oil is reported to have relaxing, anti-inflammatory, and antimicrobial properties (Valussi et al. 2021). It consists of a volatile fraction rich in limonene [DTXSID2029612], linalool, linalyl acetate, γ-terpinene [DTXSID6041210], and *β*-pinene [DTXSID7027049], and a nonvolatile fraction containing coumarins including furanocoumarins such as bergapten [also known as 5-methoxypsoralen, 5-MOP; DTXSID1025560] and bergamottin [DTXSID50897528] (Navarra et al. 2015; Ferlazzo et al. 2016; Valussi et al. 2021). Its phototoxicity effects are attributed to furanocoumarins, for example bergapten and xanthotoxin [also known as 8-methoxypsoralen, 8-MOP; DTXSID8020830] (Burnett et al. 2021). Given its widespread use, methods have been developed to remove bergapten from bergamot oil to produce non-phototoxic formulations (Gardner and McGuffin 2013).

Bergapten can cause phototoxic reactions in human skin following topical application and exposure to long-wavelength ultraviolet (UVA, 320–400 nm) radiation (Zaynoun et al. 1977; Girard et al. 1979; Gardner and McGuffin 2013). Reactions vary in erythema intensity (*i.e.,* redness caused by increased blood flow) and are often followed by marked hyperpigmentation (Zaynoun et al. 1977). In a human photo-patch test using bergamot oil containing low concentrations of bergapten, the minimum concentration capable of causing visible redness varied widely among individuals, from 6.8 ppm to 68 ppm bergapten in bergamot oil dilutions (Zaynoun et al. 1977). In the same study, the relative potencies of bergapten and xanthotoxin were evaluated, and both yielded 30% positive responses in photo patch tests at 5 ppm in ethanol and 100% positive responses at 50 ppm. In another human study, when a representative perfume containing 50 ppm bergapten was applied to the skin and followed by light exposure, mild reactions occurred in about half of the 6 participants. However, when the same treatment was applied under a UVA filtering sunscreen, no clear skin reactions were observed (Dubertret et al. 1990).

Interestingly, a recent review reevaluated the potential of several furanocoumarins, including bergapten and xanthotoxin, to cause light-induced skin reactions. The assessment identified 5 ppm as the threshold exposure concentration for furanocoumarin toxicity in leave on cosmetic products not intended for sun protection (Irizar et al. 2025).

In animal studies, bergamot oil and bergapten induced the formation of sunburn cells in guinea pig skin under UVA irradiation (Yasui and Hirone 1994). No phototoxic reaction was observed with UVB alone (Yasui and Hirone 1994). In hairless albino mice, topical bergapten enhanced solar-simulated radiation tumorigenesis, indicating photo-tumorigenic potential even at lower concentrations (Young et al. 1990).

In *in vitro* mechanistic studies, bergapten caused cytotoxicity and was mutagenic in bacteria, demonstrating its ability to induce DNA damage upon UVA exposure (Ashwood-Smith et al. 1980; Gardner and McGuffin 2013). Other experiments using cultured cells and reconstructed human skin confirmed that bergamot oils containing small amounts of bergapten caused light-induced irritation, whereas oils devoid of bergapten did not (Kejlová et al. 2007). The findings were consistent across *in vitro* and *in vivo* systems and were influenced by solvent or vehicle choice (Kejlová et al. 2007). Comprehensive *in vitro* comparisons of the photocytotoxic, photomutagenic, and photo-clastogenic effects of 16 major furanocoumarins demonstrated that bergapten exhibited the highest potency across all three endpoints (Raquet and Schrenk 2014), further supporting that bergapten is a leading contributor to bergamot oil-induced photosensitization.

Several studies investigated the use of bergamot essential oil (or constituents) for anti-inflammatory, anti-aging, or anti-acne activities (Han et al. 2017; Lombardo et al. 2020; Sun et al. 2020; Cristiano et al. 2022). These studies did not have information on skin irritation and sensitization, and other reported results are beyond the scope for this review.

Overall, bergamot essential oil with furanocoumarins is expected to cause phototoxicity, with a low potential for skin irritation and sensitization. The formulation lacking furanocoumarins is not expected to cause dermal toxicity. Both essential oils will be tested to explore potential differences in response in the NAMs.

### Parsley

Parsley (*Petroselinum crispum*, Apiaceae) is an aromatic herb native to the Mediterranean region, now cultivated worldwide. Historically valued by ancient Greek and Roman civilizations, parsley leaf was used in religious ceremonies, as a fragrance, and later as a culinary herb (Agyare et al. 2017; Punoševac et al. 2021). Roots, leaves, and seeds are all used medicinally (Agyare et al. 2017; Marthe 2020). The plant contains essential oils, flavonoids, and coumarins (including furanocoumarins). Various traditional preparations, including decoctions and infusions, have been used across regions such as Algeria, Morocco, Serbia, and Türkiye, reflecting their widespread ethnobotanical use (Šavikin et al. 2013; Güzel et al. 2015; Uzun and Kaya 2016; Bencheikh et al. 2021; Taïbi et al. 2021). Key constituents include apiole [CAS RN 523-80-8] and myristicin [DTXSID1025693], especially in the seed extracts.

Reported adverse effects from cooking or eating parsley leaf include allergic contact dermatitis, nasal and eye irritation, throat and laryngeal edema, and, in rare cases, anaphylaxis, angioedema, and urticaria (Kauppinen et al. 1980; Rademaker 1999; Janusz and Schwartz 2021). One study reported that wild parsley was responsible for an outbreak of phytophotodermatitis in 44 people (Bellringer 1949). There is a case report of skin irritation to parsley in a woman (Foti et al. 2011).

Allergic reactions may present themselves as itching, hives (urticaria), or generalized hypersensitivity following contact or ingestion. Phytophotodermatitis results from exposure to furanocoumarins (psoralens) in parsley, followed by sunlight, producing a phototoxic response similar to a severe sunburn, often characterized by localized erythema and hyperpigmented streaks or patches (Grosu Dumitrescu et al. 2024).

While we cannot access the original study, there is a report of photodermatitis in pigs exposed to parsley (Griffiths and Douglas 2000). Another older study that we cannot access reports in the abstract that several fruits and vegetables, including parsley, cause allergic responses (though it was not the top inducer) (Hannuksela and Lahti 1977).

Several studies look at using parsley for treating skin conditions (Khosravan et al. 2017). For example, a study using ointment parsley leaves extract reported a decrease in dermatitis in human studies (Ganea et al. 2024). A full review of these is beyond the scope of this review.

Overall, parsley oil extract has been shown to induce skin irritation, sensitization, and phototoxicity. Differences in responses are likely due to the part of the plant used and formula preparation. Based on these results, we anticipate that parsley will elicit dermal irritation, sensitization, and phototoxicity in the selected assays.’

### Poison ivy

Poison ivy (*Toxicodendron radicans*) is a woody perennial vine of the Anacardiaceae family, native to North America and found in parts of Asia, Central America, and the Caribbean (Innes 2012; WFO 2025). It grows in wooded or disturbed habitats and is recognized by its compound leaves of three leaflets. The plant produces an oleoresin containing urushiols, a mixture of molecules containing a catechol moiety linked to C15–C17 alk(en)yl side chains that covalently bind to skin proteins resulting in T-cell–mediated allergic contact dermatitis (Gelber et al. 1997).

Most toxicological evidence comes from accidental human exposure, clinical reports, and experimental sensitization studies. Early research demonstrated urushiol’s potent sensitization capacity: dermal application caused erythema within 48 h in humans and animals (McNair 1923; Simon 1936). Cross-reactivity among *Toxicodendron* species and other species of the family Anacardiaceae has been documented (Gaillard 1950). Urushiol’s potency varies by chemical composition (Watson et al. 1983), and *in vitro* studies confirmed activation of both CD4+ and CD8+ T-cell pathways (Kalish et al. 1994).

Recent animal studies have further clarified mechanisms involving T helper 17 cells-mediated inflammation and cytokine release, including interleukin-19 and interleukin-22 (Kim et al. 2016; Liu et al. 2019; Rashid et al. 2019). Modern non-animal approaches, such as the DPRA, confirm urushiol’s strong covalent binding activity to skin proteins (Gao et al. 2024), supporting its classification as a potent skin sensitizer. No data are yet available from other OECD-adopted NAMs such as KeratinoSens^™^ or h-CLAT, but poison ivy remains a valuable model botanical for evaluating dermal sensitization and mechanistic pathways.

Poison ivy is a well-known sensitizer. While the scientific literature is sparser than for other botanicals, this is likely due to its lack of potential medicinal use and the broad public and clinical awareness of its reactions, which may have limited research interest. Based on these results, we anticipate seeing effects in the selected sensitization assays.

### Thyme

Members of the thyme genus *Thymus* (Lamiaceae) are small, perennial, evergreen herbs with a characteristic aroma. The Working Group selected *T. zygis*, commonly known as Spanish thyme, to represent the genus in the dermal assays. Spanish thyme is native to the Mediterranean region and grows in dry, sunny climates on calcareous or sandy soils. For our review, literature on similar species, including *T. vulgaris*, was also reviewed. Historically, thyme has been used both as a culinary spice and as a medicinal herb. Traditional medicinal uses include its use as an expectorant to relieve cough and congestion, as well as an antiseptic and antifungal agent for skin and oral care (Thosar et al. 2013; Kowalczyk et al. 2020). The pharmacological activity of thyme is largely attributed to its essential oil, a product of steam distillation of the flowering aerial parts of one or several species, containing the phenolic monoterpenes thymol [DTXSID6034972] and carvacrol [DTXSID6042074]. Other volatile constituents include γ-terpinene, p-cymene [DTXSID3026645], linalool, *β*-myrcene [DTXSID6025692], and terpinen-4-ol [DTXSID4044824].

Thymol and carvacrol have moderate acute oral toxicity in rodents (LD_50_ ∼1000 mg/kg bw) and are recognized skin irritants at high concentrations (Rojas-Armas et al. 2019). In topical preparations, both are deemed safe up to 0.5% in cosmetics (CIR Expert Panel, as cited in Kowalczyk et al. (2020)). However, undiluted essential oils or concentrated extracts can cause skin irritation and allergic contact dermatitis (Uter et al. 2010; Sindle and Martin 2021). Components such as linalool and *α*-terpinene are classified as skin sensitizers and may oxidize to more potent allergenic forms upon exposure to air.

Occupational exposures to thyme dust have been reported to cause airborne contact dermatitis in agricultural workers (Spiewak et al. 2001). In a study of 46 farmers handling dried thyme, four developed erythematous skin reactions within 5–30 min of exposure, although specific immunoglobulin E reactivity was inconsistent across symptomatic and asymptomatic individuals (Spiewak et al. 2001). These findings suggest that mechanical irritation or non-immunologic mechanisms may play a role alongside occasional allergic responses.

Carvacrol and thymol have been shown to activate transient receptor potential channels, which are involved in skin sensitization and irritation (Xu et al. 2006). Thymol’s irritant and sensitizing properties have been linked to these receptor interactions, as well as to its lipophilic capacity to disrupt cell membranes (Xu et al. 2006).

Thyme essential oil is expected to induce irritation in the selected assays and may also cause sensitization, as the concentrated oil is rich in monoterpenes with strong biological activity.

## Conclusions

Botanicals are used globally in consumer products, creating a need to evaluate their potential dermal toxicity, to help ensure product safety. The BSC is examining how NAMs can be applied to screen botanicals for adverse dermal effects. The NAMs described in this review may offer a more efficient and human-relevant alternative to traditional animal toxicity testing methods for endpoints such as skin irritation, sensitization, and phototoxicity. A cross-sector team of experts has selected multiple assays to evaluate their suitability for assessing complex botanical mixtures. An important component of this effort is the use of relevant and data-rich botanicals as case studies. Careful selection of such materials is fundamental to a robust evaluation because it supports interpretation of assay performance and strengthens confidence.

This narrative review outlines the proposed assays and botanicals that were considered for evaluating dermal toxicity potential. The next steps include testing the selected botanicals, analyzing and comparing the results to existing literature, and developing an assay-based toolkit to evaluate potential to support dermal toxicity screening for botanicals. In addition to the test methods presented in this manuscript, other assays may be considered to further support results, overcome assay limitations, and provide enhanced compatibility with complex mixtures (e.g., RhE models for skin sensitization and phototoxicity or additional assays included in OECD GL 497, such as EpiSensA, ADRA, and GARDskin, which may have advantages for testing botanicals). The proposed evaluation strategy (Figure 1) includes assays that screen for irritation, phototoxicity, and sensitization and may support a future framework for assessing the applicability of established NAMs to complex botanical mixtures.

Although the current effort emphasizes evaluating the utility of *in vitro* tools for assessing the potential hazard of complex botanicals, future work may involve improving the predictive value of NAMs for human responses. Consideration of toxicokinetic factors is critical (*e.g.,* absorption through the skin, metabolism of botanical constituents, and distribution to target tissues). Bridging NAMs and *in vivo* data will help establish human-relevant exposure levels and dose-response relationships. Incorporating more advanced skin models with multiple cell types and integrated barrier functions could further refine understanding of the mechanisms underlying dermal effects.

Overall, this initiative provides a proposed strategy that may be used to develop a framework for assessing assays to evaluate the dermal toxicity of botanicals. These studies aim to enhance the botanical safety toolkit, enabling researchers to better identify dermal hazards, prioritize further testing, and improve understanding of the safety profiles of botanicals.

## Data Availability

There are no data associated with this manuscript.
